# The complete mitochondrial genome of *Alpheus digitalis* De Haan, 1844 (Decapoda: Alpheidae)

**DOI:** 10.1080/23802359.2026.2637244

**Published:** 2026-02-28

**Authors:** Shuyi Zhang, Youling Ye, Heshan Lin

**Affiliations:** Third Institute of Oceanology, Ministry of Natural Resources, Xiamen, China

**Keywords:** Mitogenomics, snapping shrimp, *Alpheus digitalis*, phylogenys

## Abstract

The snapping shrimp *Alpheus digitalis* De Haan, 1844 represents a commercially valuable fishery resource within the family Alpheidae. In this study, we sequenced and characterized the complete mitochondrial genome of *A. digitalis*, followed by phylogenetic reconstruction to elucidate its evolutionary relationships. The genome is 15,735 bp in length and contains 13 protein-coding genes (PCGs), 22 transfer RNA genes (tRNAs), 2 ribosomal RNA genes (rRNAs), and 1 D-Loop control regions, with a pronounced A + T bias (60.66%). Phylogenetic analysis based on 13 PCGs robustly supports a sister-group relationship between *A. digitalis* and *Alpheus hoplocheles*. These findings furnish essential mitogenomic resources for advancing phylogenomic, taxonomic, and evolutionary investigations of caridean shrimps.

## Introduction

The snapping shrimp *Alpheus digitalis* De Haan, 1844, classified under the order Decapoda, infraorder Caridea, family Alpheidae, is an taxonomically and ecologically significant marine crustacean (Sha et al. 2019). The genus *Alpheus* represents one of the most diverse and ecologically significant groups, widely distributed in tropical and subtropical shallow marine waters, and exhibits high heterogeneity in morphology, ecology, and behavior (Nomura and Anker 2005; Soledade et al. 2017; Zhong et al. 2019). Controversy surrounds the phylogenetic relationships within *Alpheus*, particularly whether certain clades represent cryptic species complexes or distinct lineages (Wang et al. 2020). Further evidence is needed to resolve its taxonomic status beyond traditional morphology (Williams et al. 2001; Hurt et al. 2021). Mitochondrial genomes enable detailed examination of evolutionary dynamics among decapods (Shen et al. 2012). However, complete mitochondrial data are available for only a few *Alpheus* species in public databases (Zhong et al. 2019; Yang et al. 2025), and adequate genetic information about the genus remains limited. Notably, taxonomic ambiguity has been noted between *A. digitalis* and its close relative *Alpheus hoplocheles*, partly due to morphological similarities and limited molecular data (Williams et al. 2001; Wang et al. 2020). In this study, we identified the complete mitochondrial genome of *A. digitalis* and performed phylogenetic analyses to elucidate its position within the genus.

## Materials and methods

**Figure 1.**
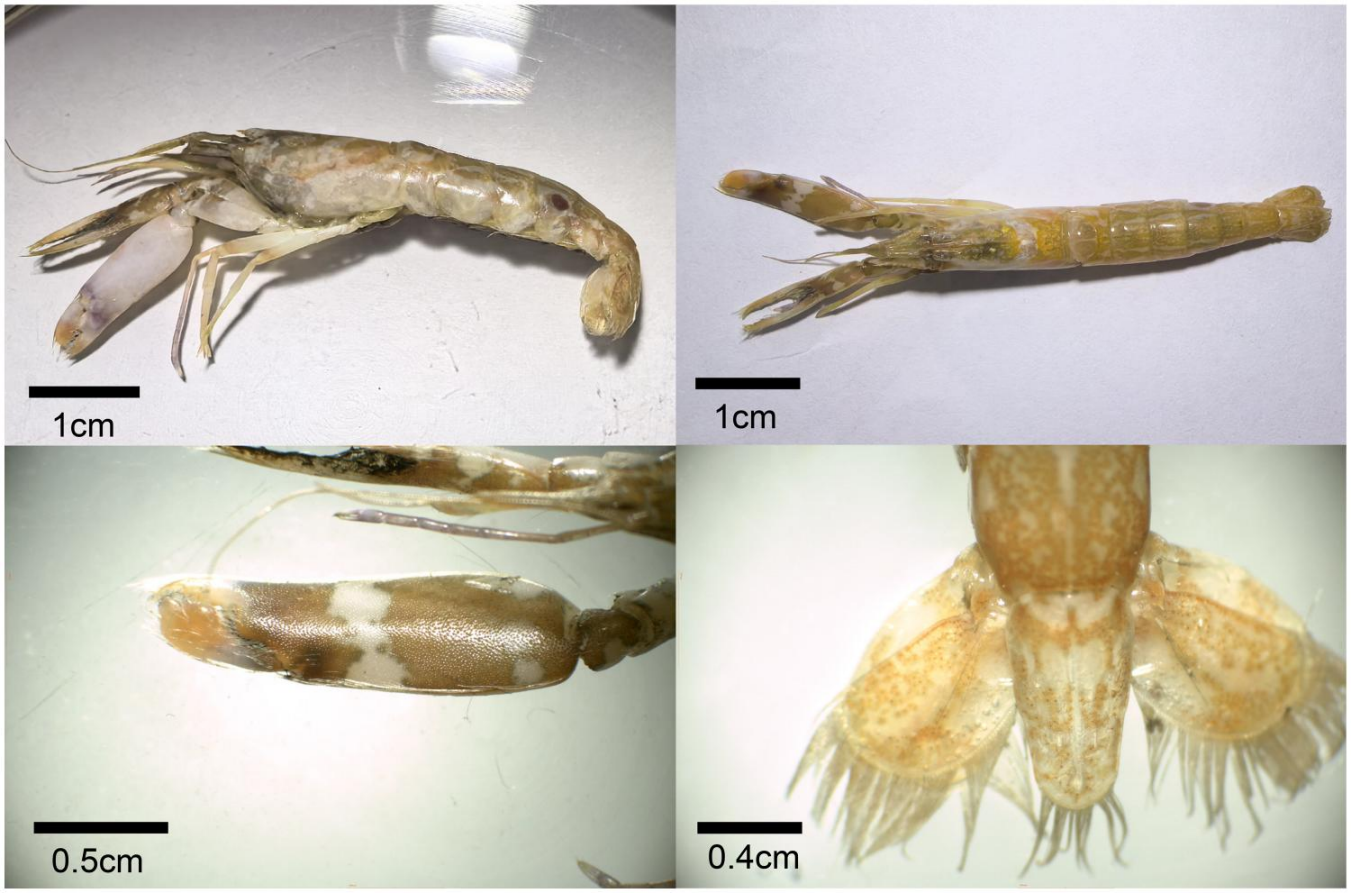
Specimen photograph for *Alpheus digitalis*. The images were photographed by Shuyi Zhang.

The specimen of *Alpheus digitali*s was collected from Xinghua Bay, Fujian Province, China (25°23.99′N, 119°26.64′E), on 25 November 2023 (Figure 1). The specimen was stored at −80 °C until processing. For DNA extraction, it was thawed, and genomic DNA was isolated from leg tissue. Following extraction, the voucher specimen was preserved in 70% ethanol. The specimen was deposited at the Third Institute of Oceanography, Ministry of Natural Resources (https://www.tio.org.cn/OWUP/index.html, Sujing Fu, fusujing@tio.org.cn) under the voucher number TIO-DAAD2023001. The total genomic DNA was extracted from pleonal muscle tissue of the specimen using an Universal Genomic DNA Kit (CoWin Biosciences, China) following the manufacturer’s protocol. DNA samples underwent gel electrophoresis, and their integrity was assessed with an automatic gel imaging analyzer. DNA libraries were constructed and sequenced on the Illumina NovaSeq 6000 platform (PE150).

Raw sequencing data were trimmed using fastp v0.23.2 (Chen et al. 2018) to remove adapter sequences and low-quality reads. A total of 21,303,800 paired-end reads were assembled into the mitochondrial genome of *A. digitalis* using SPAdes v3.15.2 (Prjibelski et al. 2020). The resulting circular mitochondrial genome was annotated using the MITOS2 WebServer (Bernt et al. 2013/). Annotations were validated by identifying PCGs using ORF Finder and BLASTX, rRNAs with Barrnap v0.9 (available at https://github.com/tseemann/barrnap), and tRNAs with tRNAscan-SE v2.0 (Lowe and Chan 2016). The mitochondrial genome map was visualized using OGDRAW v1.3.1 (Greiner et al. 2019).

A maximum-likelihood (ML) phylogenetic tree was inferred using complete mitochondrial genome from six Alpheidae species, with one Pandalidae species as the outgroup. Thirteen mitochondrial PCGs were extracted from all seven species and multiple alignment analysis was performed with MAFFT v7.310 (Katoh and Standley 2013). Poorly aligned regions were trimmed using Gblocks v0.91b (Talavera and Castresana 2007), and all alignments were combined into one supergene. The ModelFinder v2.2.0 (Kalyaanamoorthy et al. 2017) was used to identify the best suitable model of the trimmed alignment, and the ML tree was generated using IQ-TREE v2.2.0 (Nguyen et al. 2015) with 1000 bootstrap replicates. To further validate the short branch separating *A. digitalis* and *A. hoplocheles*, we additionally performed a Bayesian Inference (BI) analysis using MrBayes v3.2.7 (Ronquist et al. 2012). Two independent runs were carried out with four Markov chain Monte Carlo (MCMC) chains for 2 × 10^6^ generations, with sampling every 100 generations. The initial 25% of the runs were discarded as burn-in. The phylogenetic tree was visualized using the online tool iTOL v7 (Letunic and Bork 2007). Pairwise nucleotide differences in the 13 concatenated PCGs between *A. digitalis* and its closest relative *A. hoplocheles* were calculated using MEGAX (Kumar et al. 2018).

## Results

**Figure 2.**
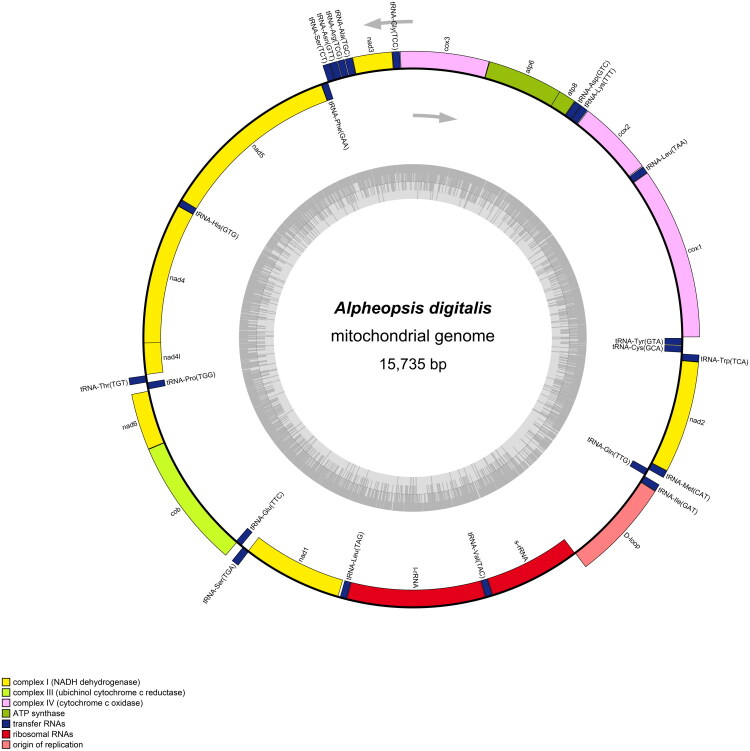
The mitochondrial genome of *Alpheus digitalis*. Annotated genes are colored according to the functional categories. Genes on the outside are transcribed in the clockwise direction, whereas genes on the inside are transcribed in the counterclockwise direction.

**Figure 3.**
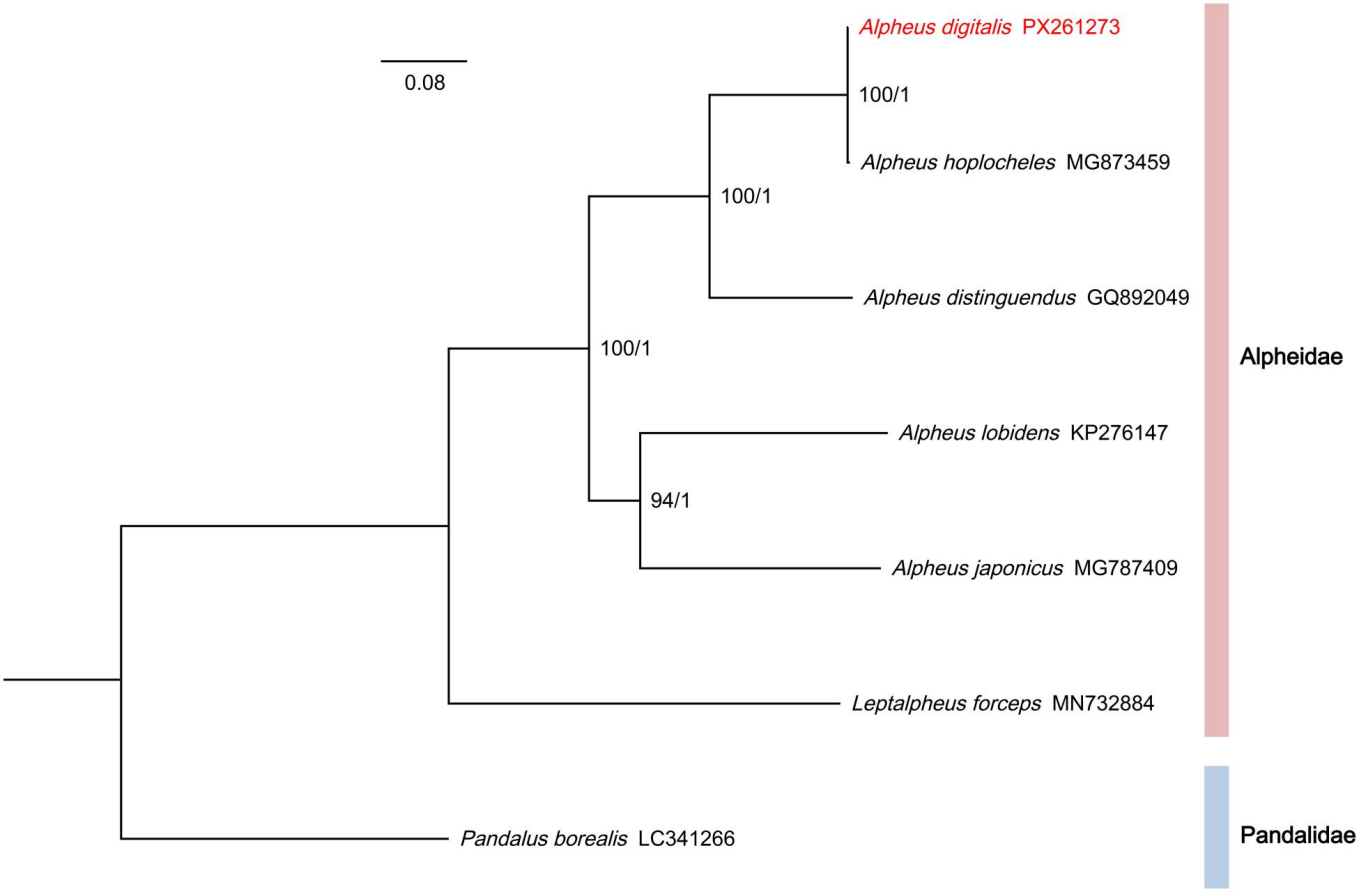
Phylogenetic tree inferred from 13 mitochondrial PCGs based on the Bayesian and maximum-likelihood methods. The numbers near each node are maximum likelihood bootstrap support values based on 1000 ultrafast bootstrap replicates in IQ-tree and Bayesian inference posterior probabilities. The position of *Alpheus digitalis* was highlighted in red. The GenBank accession numbers of mitochondrial genomes used in this analysis are the following: *Alpheus hoplocheles* MG873459 (Zhong et al. 2019), *Alpheus distinguendus* GQ892049 (Qian et al. 2011), *Alpheus lobidens* KP276147 (Wang et al. 2020), *Alpheus japonicus* MG787409 (Shen et al. 2012), *Leptalpheus forceps* MN732884 (Scioli et al. 2020), *Pandalus borealis* LC341266 (Xu et al. 2018).

The mitochondrial genome of *A. digitalis* was 15,735 bp in length (Figure 2), and the average read mapping depth was 129× (Figure S1). The genome encoded the typical set of 37 metazoan genes, including 13 PCGs, 22 tRNAs, 2 rRNAs, and 1 D-Loop control regions (Figure 2). Structurally, the mitochondrial genome of *A. digitalis* exhibits a compact organization, with the largest intergenic spacer measuring only 19 bp between *tRNA-Glu* and *tRNA-Ser*. Additionally, the entire genome had 10 overlapping regions, each ranging from 1 to 16 bp in length. The overall base composition of the mitochondrial genome was estimated to be A 33.14%, T 27.52%, C 25.61%, and G 13.73%, with a high A + T content of 60.66%, which is within the range of A + T content of published alpheid mitochondrial genome (Qian et al. 2011; Shen et al. 2012). Nine PCGs (namely, *nad2*, *nad3*, *nad6*, *atp6*, *atp8*, *cox1*, *cox2*, *cox3*, and *cob*) were encoded on the heavy strand, while the remaining four genes (namely, nad1, nad4, nad4l, and nad5) were encoded on the light strand. Our phylogenetic analysis revealed that *A. digitalis* and *A. hoplocheles* share the closest evolutionary ties and form a sister group (Figure 3). Notably, the branch separating these two species was the shortest in the entire tree, yet their sister-group relationship received maximal support in both maximum-likelihood and Bayesian inference analyses (Figure S2). Direct comparison of the 13 concatenated protein-coding genes revealed 22 nucleotide differences between *A. digitalis* and *A. hoplocheles*.

## Discussion and conclusion

This study presents the first complete mitochondrial genome of *A. digitalis*, which exhibits a typical circular structure and a high A + T content (60.66%), consistent with previously reported alpheids (Qian et al. 2011; Shen et al. 2012). The compact architecture, with limited intergenic spacers and multiple overlapping regions, reflects evolutionary constraints common in decapod mitochondrial genomes (Kilpert and Podsiadlowski 2006; Shen et al. 2013). Phylogenetic analysis based on 13 PCGs strongly supports a sister relationship between *A. digitalis* and *A. hoplocheles*, supporting previous hypotheses of close evolutionary relationships within certain *Alpheus* clades (Wang et al. 2020). The maximal statistical support for the sister-group relationship between *A. digitalis* and *A. hoplocheles*, despite the shortest branch lengths in the tree, is consistent with a very recent divergence event. Although genetic divergence is minimal, the 22 nucleotide differences in the 13 PCGs and the clear resolution of terminal branches provide robust molecular evidence for recognizing *A. digitalis* and *A. hoplocheles* as distinct but very closely related species. These findings provide essential genomic resources that contribute to resolving taxonomic ambiguities and phylogenetic conflicts in Alpheidae. The mitogenome data offered here will facilitate further studies on species delimitation, molecular evolution, and phylogenomics of caridean shrimps, particularly for understudied yet ecologically significant taxa like *A. digitalis*.

## Supplementary Material

Supplementary materials.pdf

Supplementary Figure Legend.docx

## Data Availability

The genome sequence data that support the findings of this study are openly available in GenBank of NCBI at [https://www.ncbi.nlm.nih.gov] under the accession no. PX261273. The associated BioProject, BioSample and SRA numbers are PRJNA1327781, SAMN51292689, and SRR35361759, respectively.
