# Supplementary material for: The complete mitochondrial genome of Alpheus digitalis De Haan, 1844 (Decapoda: Alpheidae)
Source: Mitochondrial DNA B Resour. 2026 Feb 28;11(4):478–82. doi: 10.1080/23802359.2026.2637244 (PMC12951682; doi:10.1080/23802359.2026.2637244)
Supplement: Supplementary Figure Legend.docx [file TMDN_A_2637244_SM2289.docx]

**Supplementary Figure Legend**

Figure S1. The estimated coverage depths from Illumina sequencing reads for the mitochondrial genome of *Alpheus digitali*s.

Figure S2. Phylogenetic tree inferred from the partitioned nucleotide sequences of 13 mitochondrial PCGs based on the Bayesian inference methods. The numbers near each node is Bayesian inference posterior probabilities.
